# Exchange bias effect in polycrystalline Bi_0.5_Sr_0.5_Fe_0.5_Cr_0.5_O_3_ bulk

**DOI:** 10.1038/s41598-023-32734-x

**Published:** 2023-04-18

**Authors:** S. Z. Li, A. Rahman, C. L. Ma, X. Zhao, Z. Y. Sun, M. F. Liu, X. Z. Wang, X. F. Xu, J. M. Liu

**Affiliations:** 1 School of Electrical and Electronic Engineering, Wuhan Polytechnic University, Wuhan, 430048 China; 2 Department of Physics, University of Science and Technology of China, Hefei, 230026 China; 3 Jiangsu Key Laboratory of Micro and Nano Heat Fluid Flow Technology and Energy Application, School of Physical Science and Technology, Suzhou University of Science and Technology, Suzhou, 215009 China; 4 Institute for Advanced Materials, Hubei Normal University, Huangshi, 435002 China; 5 Institution of Quatum Material, Hubei Polytechnic University, Huangshi, 435003 China; 6 Nanjing National Laboratory of Microstructure, Nanjing University, Nanjing, 210093 China

**Keywords:** Materials science, Physics

## Abstract

Bulk Bi_0.5_Sr_0.5_Fe_0.5_Cr_0.5_O_3_ (BSFCO) is a new compound comprising the *R3c* structure. The structural, magnetic property and exchange bias (EB) details are investigated. The material was in the super-paramagnetic (SP) state at room temperature. Exchange bias usually occurs at the boundary between different magnetic states after field cooling (*H*_*FC*_) acts on the sample. Here the result shows that changing *H*_*FC*_ from 1 to 6 T reduces the *H*_*EB*_ value by 16% at 2 K at the same time. Meanwhile, *H*_*EB*_ diminishes as the ferromagnetic layer thickness increases. The variation of (the thickness of ferromagnetic layer) *t*_FM_ with the change of *H*_FC_ leads to the tuning of *H*_EB_ by *H*_FC_ in BSFCO bulk. These effects are obviously different from the phenomenon seen in other oxide types.

## Introduction

Exchange coupling often occurs at the interface between the ferromagnetic (FM) and antiferromagnetic (AF) layers. When a magnetic field *(H)* is applied, once the sample temperature is below the AF layer Néel temperature, the FM layer exhibits unidirectional anisotropy because of the exchange coupling^1,2^. As a result, the hysteresis loop shifts along the field axis. The strength of the shift is called by the exchange bias (EB). The EB effect is attributed to a FM unidirectional anisotropy formed at the interface between different magnetic phased^3^. There are some EB effects in artificial material systems, for example, FM-AFM bilayers, FM-AFM superlattices, and topological-insulator/antiferromagnet containing ferromagnetic antiferromagnetic (FM-AFM) components^4–11^.

Up to now, some progress has been made in the investigation of man-made EB structures. However, there are still some issues with the use of artificial structures that have been modified with various material phases. The artificial EB usually benefits from field-cooling process. But artificial material with chaotic ionic diffusion at the interface maybe influences on the FM-AFM coupling. In recent years, investigations have demonstrated unusual EB under field-cooling (FC) and large spontaneous EB in a variety of systems with a single phase, including BiFeO_3_–Bi_2_Fe_4_O_9_ nanocomposite, NiMnIn, Mn_2_PtGa, La_1.5_Sr_0.5_MnCoO_6_, Pb_6_Ni_9_(TeO_6_)_5_, Y_0.2_Ca_0.8_MnO_3_, and Bi_10_Fe_6_Ti_3_O_30_ polycrystalline samples^6,12–17^.

From above, materials with EB effect in a single phase should be investigated further to better understand the mechanism. Baettig and Spaldin performed first-principles calculations on the BiFeO_3_–BiCrO_3_ system^18^. A double-perovskite Bi_2_FeCrO_6_ with a long-range Fe^3+^–Cr^3+^ order was hypothetically constructed, and a magnetic moment of 2 µB per formula unit were predicted ^18^. Experiments focused on the substituting effect of Cr ion for Fe ion on the physical properties of BFO^19^. But in bulk, it is difficult to prepare, so SrCrO_3_ and Bi(Fe,Cr)O_3_ must be both prepared under high pressure^20^. For BFO, Bi(Fe,Cr)O_3_ and SrCrO_3_ bulk, appearance of weak FM could result in glassy state between AFM and FM interactions. Therefore, we investigate the Bi_0.5_Sr_0.5_Fe_0.5_Cr_0.5_O_3_ system in search of stable perovskite phases that may exhibit some of the above properties. Here the structure and magnetic properties of the new phase Bi_0.5_Sr_0.5_Fe_0.5_Cr_0.5_O_3_ under ambient pressure. And we present the observation FM, AFM and SP in the BSFCO bulk and discuss the role of multiple of magnetic phases on EB effects. The results show that Bi_0.5_Sr_0.5_Fe_0.5_Cr_0.5_O_3_ bulk has the potential to be a new class material, which could help to improve magnetic tuning.

## Experiments

Bi_0.5_Sr_0.5_Fe_0.5_Cr_0.5_O_3_ (BSFCO) bulk was prepared by solid reaction. High-purity SrCO_3_, Bi_2_O_3_ (10% excess), Fe_2_O_3_, and Cr_2_O_3_ were intimately synthesized for 24 h, in stoichiometric quantities with solid reaction method under flowing nitrogen at 950 °C and 1100 °C. X-ray powder diffraction supplied the formation of a single perovskite type phase. Measurements of the crystal structure of the samples were performed on a ‘Rigaku UltimaIV’ X-ray diffraction apparatus (XRD, 285 mm 3 kW and CuKα1). The data were analyzed by using the GSAS suite of Rietveld analysis programs. A superconducting quantum interference device magnetometer (SQUID, Quantum Design Inc.) was employed to carry out magnetization measurements. After undergoing zero field cooling (ZFC) and field cooling (FC), measurements were conducted when the sample was heated from 5 to 300 K under* H* = 10 kOe. A magnetization loop was measured at 2 K. Mössbauer data were obtained using a conventional constant acceleration Mössbauer spectrometer incorporating a 25 mCi source of ^57^Co in an Rh matrix. The valence states of Fe ions and Cr ions in bulk BSFCO were detected by the X-ray photoelectron spectroscopy (Al kα source, h*ν* = 1.486 eV).

## Results

### XRD patterns

**Figure 1:**
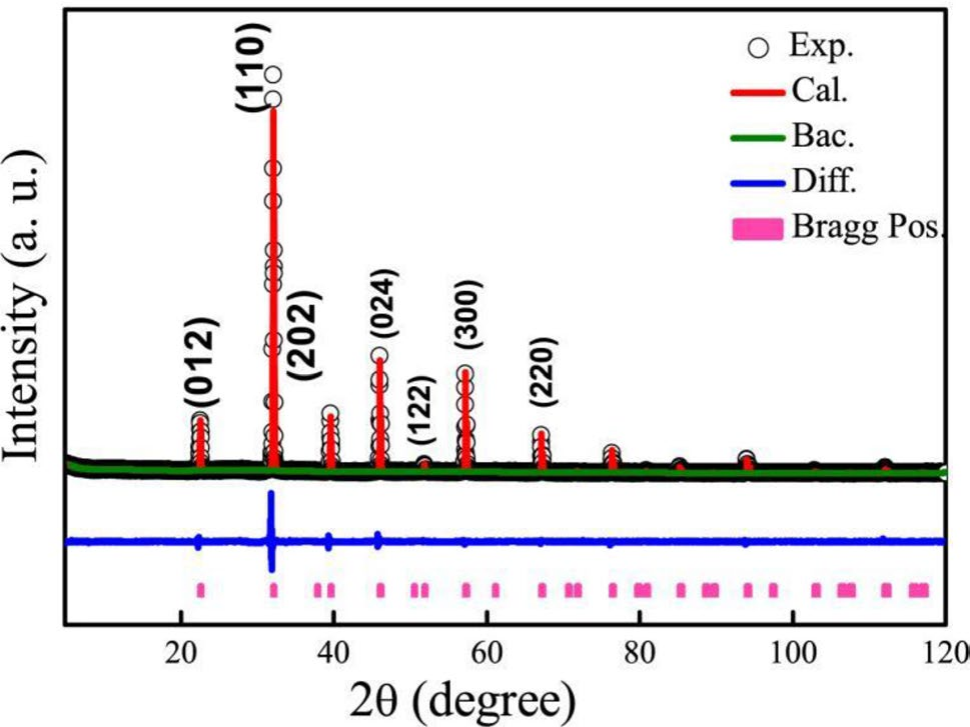
X-ray diffraction patterns for BSFCO are shown at the room temperature. The refined structure diffraction data are observed together with the experimental data points using Gasa refinement.

Figure 1 depicts the XRD patterns of Bi_0.5_Sr_0.5_Fe_0.5_Cr_0.5_O_3_ at room temperature. The diffraction data reveals the same structure with a hexagonal *R3c* unit cell, which is similar with BiFeO_3_ previously reported^20^. Since Sr ionic has a smaller size than that of Ba ion and the ionic size of Cr is smaller than that of Fe, this leads to the lattice constants change. To discuss the crystal lattice structure changes, the structure was refined using Rietveld, and the data was fitted to the experimental pattern. The lattice parameters were refined and shown with Chi^2^ = 1.877, *R*_wp_ = 5.97%, *R*_p_ = 4.58% in Table 1. The cell parameters of sample were *a* = 5.5726(22) Å, *b* = 5.5726(22) Å, *c* = 13.6500(64) Å. The site of the cell is *x*, *y* and *z* in Table 1. It is found that the lattice constant that *a* and *c* decrease because Sr and Cr substitute Bi and Fe, respectively. The in-plane lattice parameter *a* is reduced to 5.572 Å, and the out-of-plane lattice parameter *c* decreases and is up to 13.6500 Å. No additional reflections and impurities are detected in the room-temperature XRD data of bulk Bi_0.5_Sr_0.5_Fe_0.5_Cr_0.5_O_3_.

**Table 1:** Refined structural parameters of Bi_0.5_Sr_0.5_Fe_0.5_Cr_0.5_O_3_ from powder diffraction data collected at room temperature.

Atom (site)	*x*	*y*	*z*	Occ	*U*_iso_
Bi1(6*a*)	0.0000	0.0000	− 0.1668(2)	0.5000	0.0866(8)
Fe1(6*a*)	0.0000	0.0000	0.0838(3)	0.5000	0.0195(9)
Sr1(6*a*)	0.0000	0.0000	− 0.1668(2)	0.5000	0.0866(8)
Cr1(6*a*)	0.0000	0.0000	0.0838(3)	0.5000	0.0195(9)
O1(18*b*)	0.5038(2)	0.5001(1)	0.8383(5)	1.0000	0.0363(5)

SP: *R*3*c*, *a* = 5.5726(22) Å, *b* = 5.5726(22) Å, *c* = 13.6500(64) Å, Chi^2^ = 1.877, *R*_wp_ = 5.97%, *R*_p_ = 4.58%.

**Figure 2:**
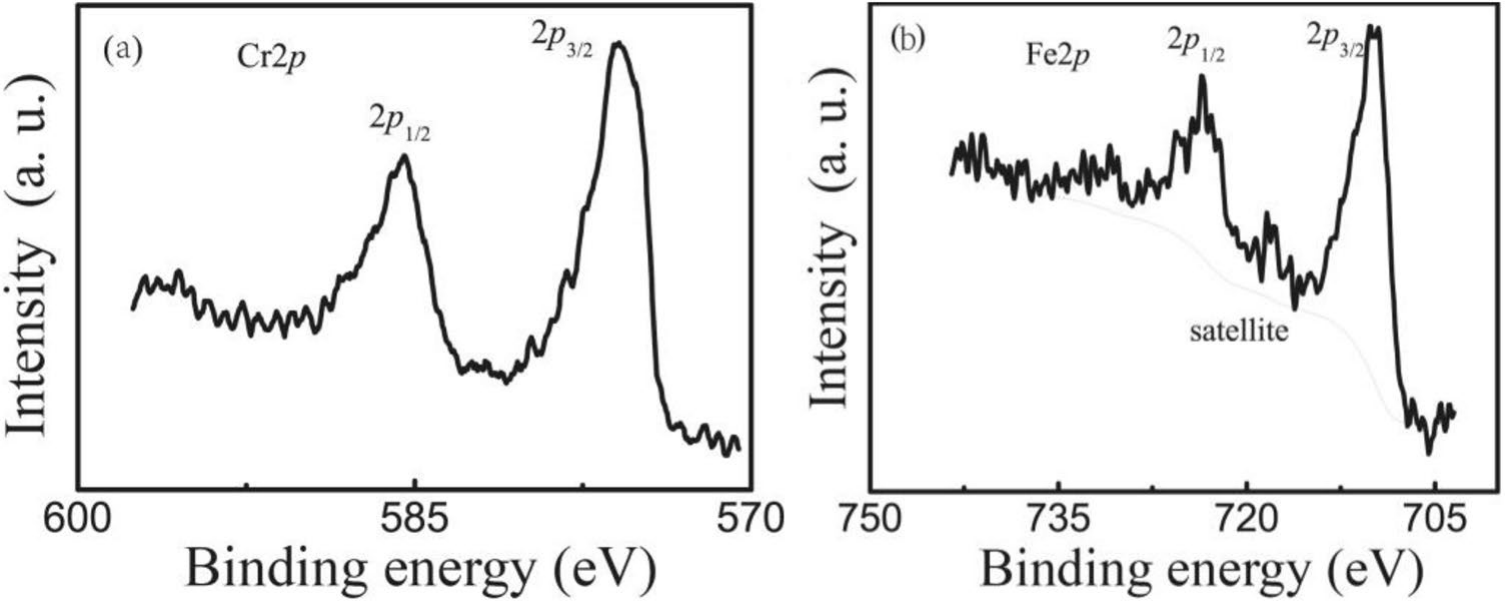
The core level XPS spectra for constituent elements in bulk BSCFO (**a**) Cr^3+^and (**b**) for Fe^3+^.

In order to get the atomic valence, Fig. 2 illustrates the Fe 2p and Cr 2p XPS spectra of bulk Bi_0.5_Sr_0.5_Fe_0.5_Cr_0.5_O_3_. Figure 2a shows the binding energies of Cr 2p. The energies 576.2 eV and 586.2 eV correspond to Cr 2p_3/2_ and Cr 2p_1/2_, respectively. It demonstrates that the Cr ions in BSFCO are mainly Cr^3+^^22^. The Fe 2p_3/2_ and Fe 2p_1/2_ peaks are at about 711.7 eV and 725.3 eV in Fig. 2b, respectively. Fe^3+^ ions have binding energies. And Fe 2p_1/2_ with a satellite is at 719.6 eV^21,22^. Therefore, it can be deduced that Fe ions in the bulk BSFCO are primarily Fe^3+^, with a few Fe^2+^ ions present to compensate for oxygen vacancies that cannot be determined.

### The Mössbauer spectrum and magnetization of bulk BSFCO

**Figure 3:**
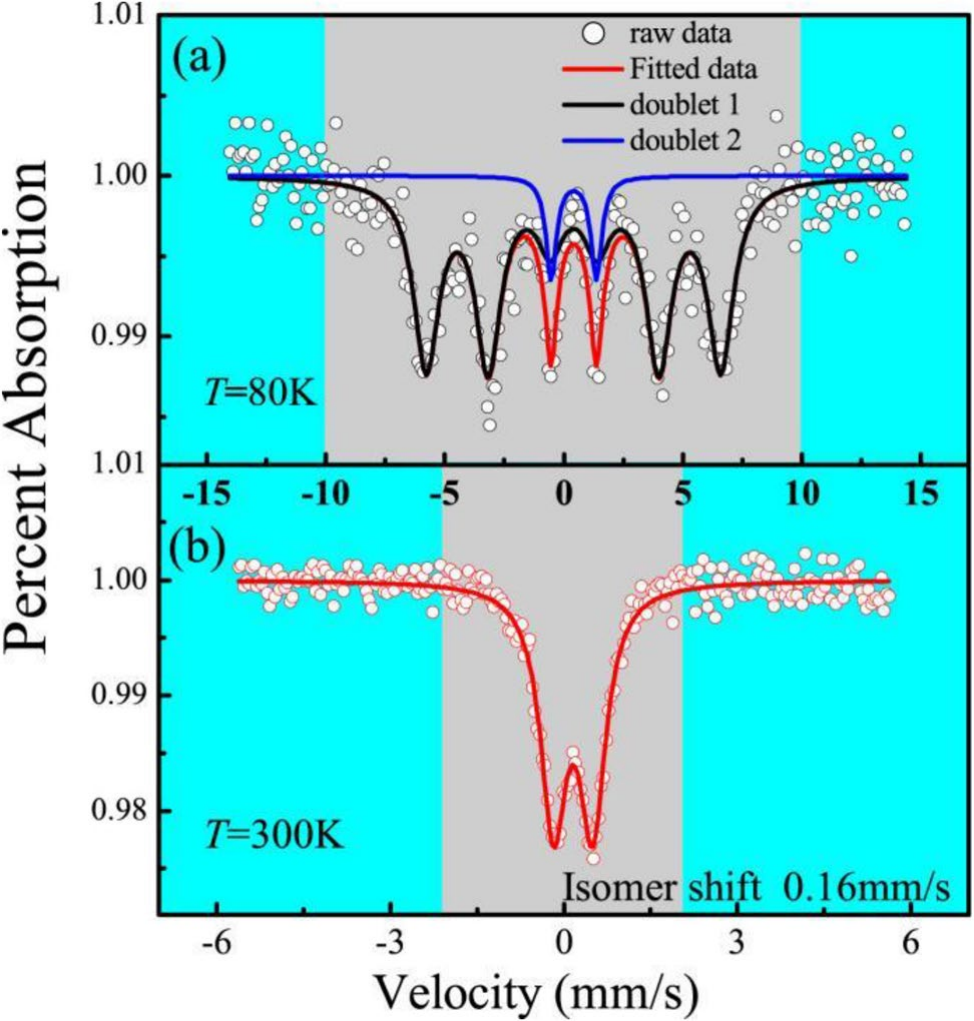
The Mössbauer data of bulk BSCFO at 80 K (**a**) and 300 K (**b**).

Mössbauer data for bulk BSFCO samples at 80 K (a) and 300 K (b) are shown in Fig. 3. Mössbauer spectroscopy is used to demonstrate the bulk magnetic behavior. The 80-K spectrum in Fig. 3a was fitted to a six-line magnetic profile (doublet 1 in black, Chi^2^ = 1.2) and a double magnetic profile (doublet 2 in blue, Chi^2^ = 0.9). In Fig. 3b, the data can be fitted to a doublet profile and shows super-paramagnetic relaxation at 300 K. All related data are shown in Table 2. At 300 K, it is a paramagnetic/antiferromagnetic material with an isomer shift of 0.16(0) mm/s. The property of quadrupole splitting indicates the presence of a distorted octahedral environment, which agrees with bond distances refined from X-ray diffraction.

**Table 2:** Mössbauer data at 300 K and 80 K for BSFCO bulk.

Temperature	*Area*	IS* (mm/s)	*QS* (mm/s)	*H (kOe)*	*W* Γ (mm/s)
300 K		0.16(0)	0.67(8)		0.54(6)
80 K	90.9%	0.40(7)		383.(3)	1.28(4)
	9.1%	0.41(7)	1.89(2)		0.54(0)

As the temperature decreases, there are strong super-exchange interactions between the metal ions, which causes the splitting of the magnetic energy levels of the nuclei, thus making the Mössbauer spectrum exhibit a magnetic splitting six-line magnetic profile and super-paramagnetic relaxation. In Fig. 3a, the quadrupole interaction at 80 K is 0.40(7) mm/s, which is the characteristc of Fe^3+^^23^. Meanwhile, the width of the spectrum at 80 K is wider than that at 300 K. The areas of doublet 1 and doublet 2 are 90.9% and 9.1% with super-magnetic relaxation, respectively. The ferromagnetic component of the spectrum becomes stronger and the paramagnetic component becomes weaker. Therefore, the paramagnetic spectrum in the sample spectra should be derived from the super-paramagnetic relaxation caused by the small size effect^24^.

**Figure 4:**
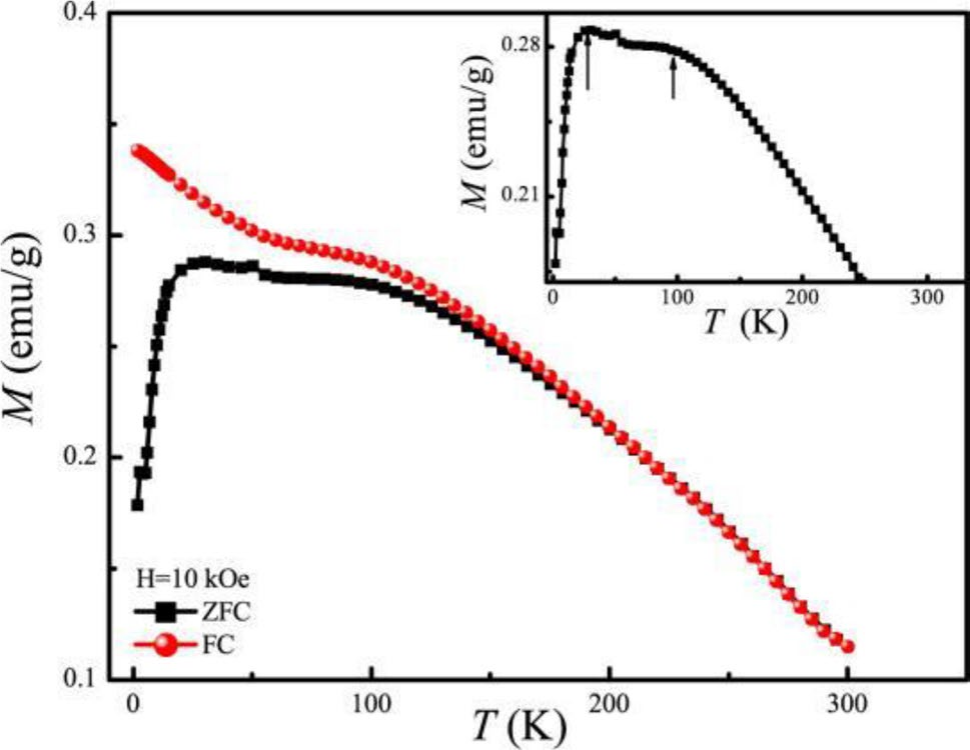
The ZFC and FC curve of BSCFO bulk under *H* = 10 kOe.

ZFC-Field cooling (FC) curves are measured to investigate the magnetization of bulk BSFCO at low temperatures as shown in Fig. 4. And enlarged ZFC curve is in the inset. Figure 4 shows the magnetization curves of bulk BSFCO at zero field and cooling field after *H* = 10 kOe and *T* = 300 K. The bulk magnetic behavior of BSFCO is revealed by magnetization measurements and plotted as a *M*-*T* curve. The ZFC curve has a plate-like peak at 25 K and the magnetization decreases rapidly below 25 K, while the ZFC and FC curves split around 130 K, which is similar to that of Ni-Mn-In bulk alloys^13^. It is an obvious feature of coexisting FM (super-ferromagnetic) cluster and an AFM matrix. The super-ferromagnetic domains are collectively frozen forming spin glass (SG) state at lower temperatures.[13]The plate-like curve suggests the existence of competing interactions among its ferromagnetic property, antiferromagnetic property and SG. Consequently, it may not be linked to spin glass-like freezing with long range order, but a transition to short range order.

### *M*–*H* properties

**Figure 5:**
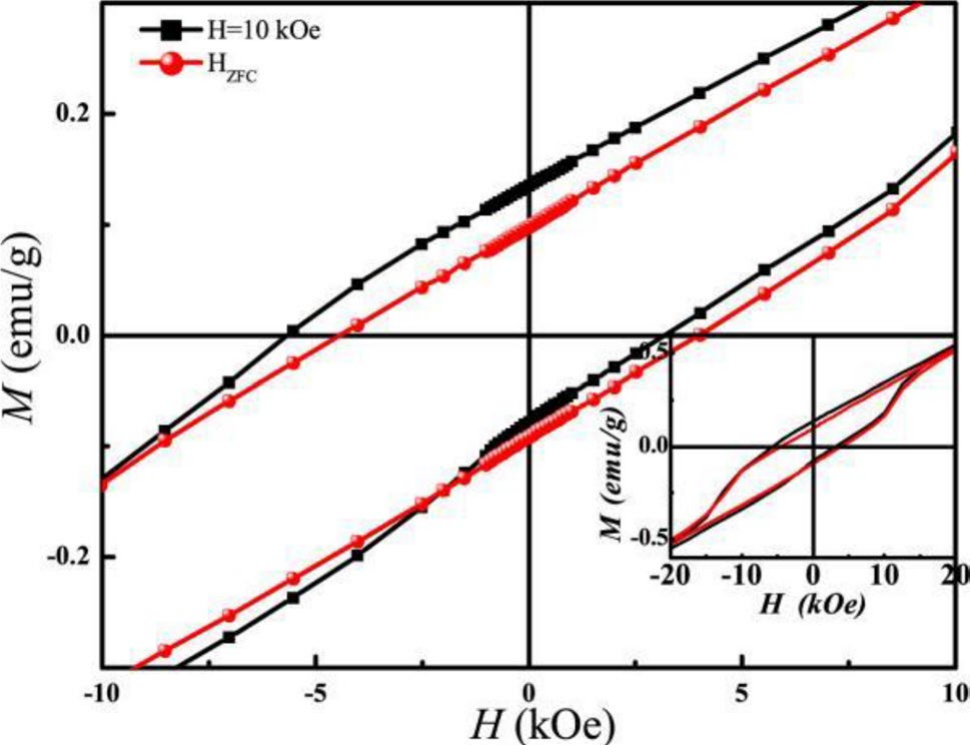
Hysteresis loops of magnetization at 2 K after ZFC and FC under *H* = 10 kOe from 300 K. The inset is the enlarged graph of hysteresis loop.

To confirm these magnetic properties, the hysteresis loops of magnetization were collected from − 10 kOe to 10 kOe at 2 K after ZFC; And the data were gotten from FC under *H* = 10 kOe decreasing from 300 K, as shown in Fig. 5. The *M*–*H* loop under ZFC state is symmetric around zero, whereas the existence of exchange bias was proved by the shift of FC loops towards negative field. *H*_*EB*_ and *H*_*C*_ parameters are defined as *H*_*EB*_ = (*H*_*1*_ + *H*_*2*_)/2 and *H*_*C*_ = − (*H*_*1*_ − *H*_*2*_)/2, respectively, where *H*_*1*_ and *H*_*2*_ are the left and right coercivity field. *H*_EB_ was about 1150 Oe under the FC condition. *H*_C_ obtained from the FC loop is about 460 Oe and it is slightly higher than *H*_C_ obtained from ZFC (410 Oe). The different values result from the function of the exchange anisotropy because the magnetic field acts or not.

**Figure 6:**
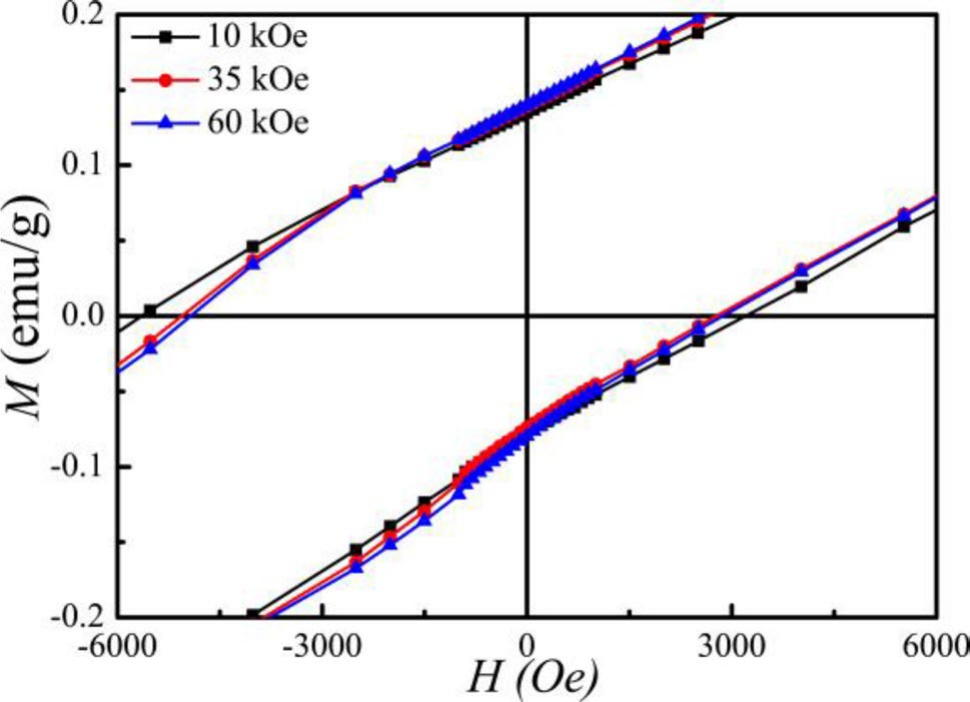
Hysteresis loops of magnetization at 2 K after FC under *H* = 10 kOe, 35 kOe and 60 kOe.

It is shown in Fig. 6, with the reduction of *H*_EB_ and *H*_C_ by the reversal of subsequent magnetization, the so-called training effect is present. This effect indicates that the exchange anisotropy slowly decreases. From the *M*-*H* loops (Fig. 6), it is observed that the ZFC magnetizations at 10 kOe, 35 kOe, and 50 kOe are much smaller than that of their FC counterparts. So FC can enlarge the content of the super-ferromagnetic (SFM) or FM region and ferromagnetic layer (*t*_FM_). The increase of *t*_FM_ under FC conditions reduces strain anisotropy, which arises from different magnetic states among the FM layer, the AF layer, and disordered spin glass; meanwhile, the strain anisotropy could cause the decrease of *H*_C_.

**Figure 7:**
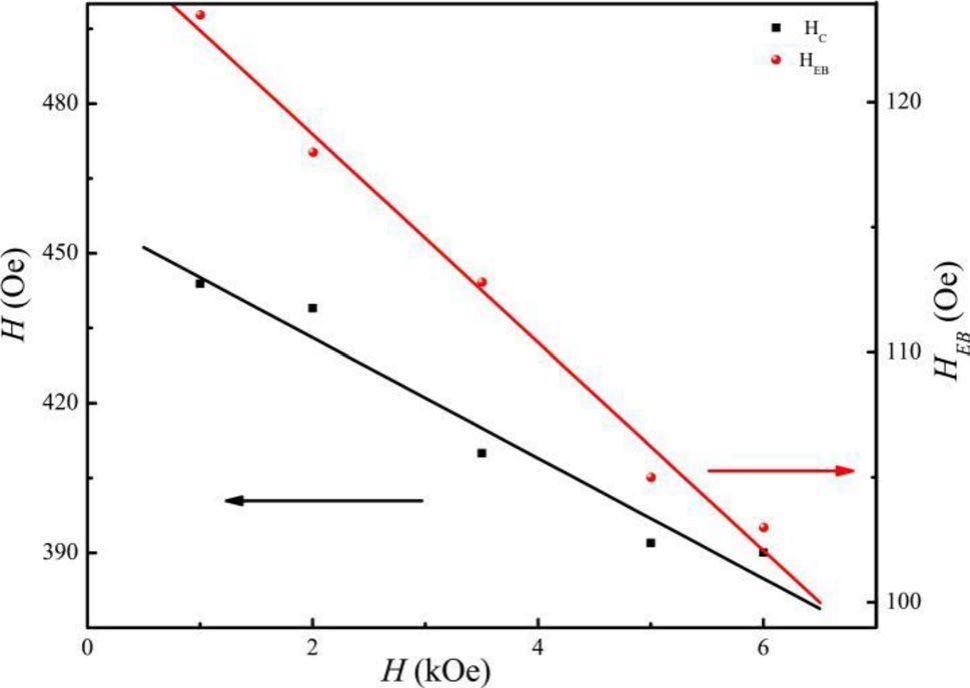
*H*_FC_ dependence of *H*_C_ (right-hand axis) and *H*_EB_ (left-hand axis) at 2 K. *H*_C_ and *H*_EB_ decrease linearly with *H*_FC_. The lines are provided as a guide to the eye.

From above, we can deduce that changing *H*_*FC*_ can affect *t*_*FM*_ and the ratio of *H*_*C*_ (*H*_c_ under FC) and *H*_*EB*_ (*H*_*EB*_ under FC), as shown in Fig. 7. When *H*_*FC*_ increases, both *H*_*C*_ and *H*_*EB*_ decrease. When *H*_*FC*_ increases from 10 to 60 kOe, *H*_*EB*_ decreases by 16% at 2 K. It is not difficult to conclude from the above analysis that strain anisotropy could decrease^16^. When the sample under FC exists, an FM-AFM interface with unidirectional FM spins formed. On one hand, it is known that AFM domains with anisotropy axis parallel to external magnetic. On the other hand, for AFM domains with anisotropy axis nonparallel to external magnetic field, there is an angle between the direction of the initial magnetization. So increasing external magnetic field, *H*_*EB*_ will decrease^10^.

**Figure 8:**
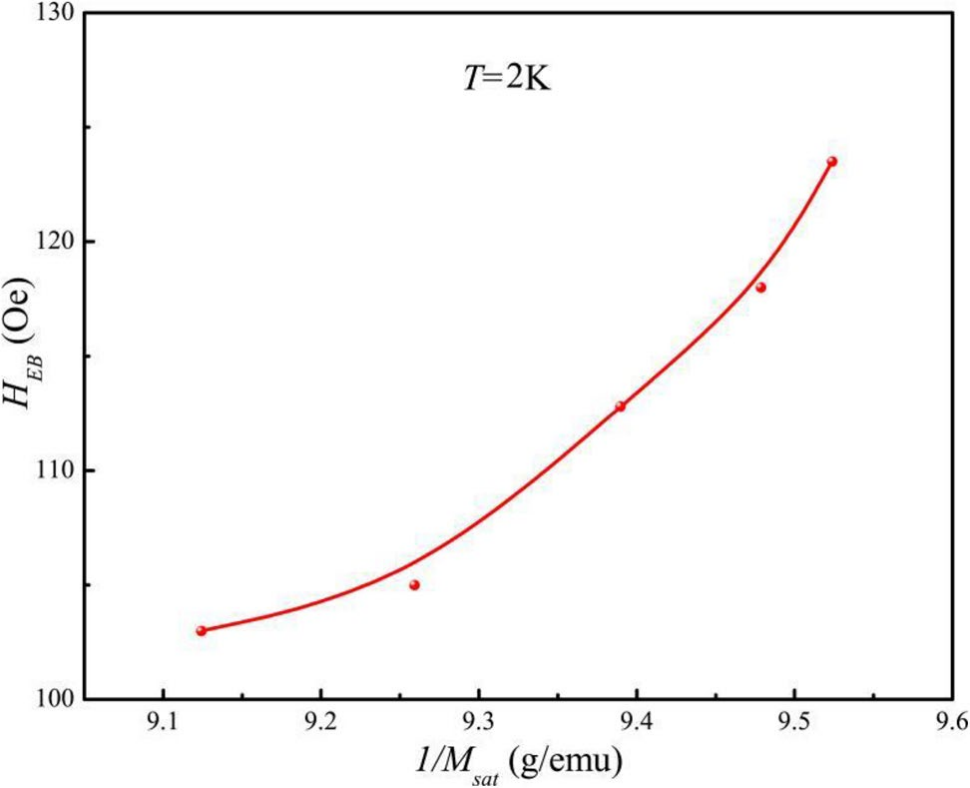
Linear relationship between *H*_EB_ and 1/*M*_sat_ at 2 K. Note that *M*_sat_ scales *t*_FM_, indicating that the exchange bias in bulk BSFCO follows *H*_EB_ ∝ 1/*t*_FM_.

To demonstrate the relationship between *t*_*FM*_ and *H*_*EB*_ in bulk BSCFO, the dependence of *t*_*FM*_ on *H*_*FC*_ must be confirmed. From the *M*-*H* loop in Fig. 6, the magnetization of the FM layers is almost observed under an applied field of 10 kOe. *M*_*sa*t_, defined as (*M*_*10kOe*_*-M-*_*10kOe*_)/2, is proportional to the volume fraction of the FM region^3^. *M*_*sat*_ increases with *H*_*FC*_ when *t*_*FM*_ emerges, so the *M*_*sat*_-*H*_*FC*_ curve in Fig. 8 scales the variation of *t*_*FM*_ with *H*_*FC*_. *H*_*EB*_ and 1/*M*_*sat*_ have a quasi-linear relationship, as illustrated in Fig. 8. *H*_*EB*_ decreases as *t*_*FM*_ increases, which is consistent with the trend in exchange bias films^3^. The result demonstrates the presence of FM coupling at the FM/AF interface in BSCFO. In the further, the AF coupling at the interface would result in a competition between the exchange energy and the Zeeman energy. It would weaken the relationship between *H*_EB_ and 1/*t*_FM_. This trend deviates from the linear prediction. It is worth noting that *H*_*FC*_ changes the anti-ferromagnetic thickness (*t*_*AF*_), which has an impact on *H*_*EB*_.

This implies that the majority of the regions in the system are AF, but *t*_*FM*_ remains constant after sample fabrication without being affected by *H*_*FC*_. The interfacial spins of SFM and/or AF layers with *H*_*FC*_ will have an effect on *H*_*EB*_ change. The spontaneous FM and AF layers in BSCFO remain constant, allowing *t*_*FM*_ to be tuned after fabrication utilizing external forces. The mutual interactions of charge, defects, spin, and lattice degrees of freedom in this bulk material can result in a delicate balance of different ground states^3,10^.

**Figure 9:**
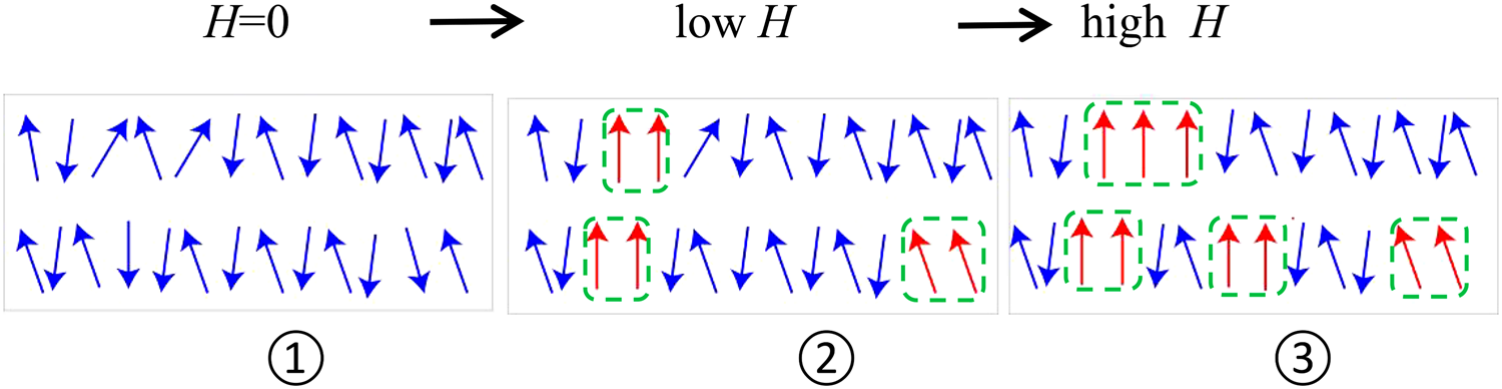
Schematic diagram of the AFM/FM structure for bulk BSCFO under different magnetic fields. The datasets generated and/or analyzed during the current study are available in the [CSD Crystallographic data] repository, [Summary of Data—Deposition Number 2151932, Compound Name:Data Block Name: data_BFSC_publ Unit Cell Parameters: a 5.57262(5) b 5.57262 c 13.65006(10) R3c.

In order to explore the above phenomenon, the evolution of the SFM/AFM spin interface of BSFCO after FC under external magnetic field was shown in Fig. 9. It is a simplified schematic diagram with SFM or FM/AFM interface embedded in an AFM domain. Wang et. al. believe that a SFM unidirectional anisotropy, which is similar to an FM unidirectional anisotropy, can be formed during the initial magnetization process^13^.The applied magnetic field aligns some of the AFM spin along the direction of external field. FM structure can be influenced with AFM by interfacial coupling, so the magnetization can be changed to a greater extent under a PM background, as shown in Fig. 9 (①②③). Because the Zeman energy of AFM spins near the interface is larger than the coupling energy of AFM/FM at the interface. Some AFM spins can aligned along the direction of external field. It is identified from above that external fields have an effect on the interaction between the AF and FM layers in Fig. 9(②③). Furthermore, it benefits FM phase enhancement, as increasing *H*_*FC*_ can increase the ration of FM content and *t*_*FM*_ while decreasing *H*_*EB*_. This indicates that, in the presence of certain external factors, the sensitivities of order parameters can be used to tune the exchange bias. Some recent studies suggest that short disorder is beneficial to FM clusters/microstructure in La_1.5_Sr_0.5_CoMnO_6_ and Mn_2_PtGa materials^6,13^. The exchange anisotropic coupling of the embedded phase between FM and AFM layers has resulted in EB. Thus, it's reasonable to assume that the EB in bulk BSFCO is due to the coupling between the AFM layer and SFM or FM layer. To summarize, the analysis of exchange bias in these types of materials may lead to unusual events, which could aid in the creation of multifunctional spintronic devices.

## Supplementary Information

Supplementary Information.

## Data Availability

All data generated or analysed during this study are included in this published article and supplementary files.
