# Supplementary material for: Exchange bias effect in polycrystalline Bi0.5Sr0.5Fe0.5Cr0.5O3 bulk
Source: Sci Rep. 2023 Apr 18;13:6333. doi: 10.1038/s41598-023-32734-x (PMC10113268; doi:10.1038/s41598-023-32734-x)

checkCIF/PLATON report

Structure factors have been supplied for datablock(s) BFSC_publ

THIS REPORT IS FOR GUIDANCE ONLY. IF USED AS PART OF A REVIEW PROCEDURE FOR PUBLICATION, IT SHOULD NOT REPLACE THE EXPERTISE OF AN EXPERIENCED CRYSTALLOGRAPHIC REFEREE.

No syntax errors found. CIF dictionary Interpreting this report

Datablock: BFSC_publ

Bond precision:	= 0.0000 A		Wavelength=1.54430
Cell:	a=5.57262(2)	b=5.57262(2)	c=13.65006(10)
	alpha=90	beta=90	gamma=120
Temperature:	0 K		

	Calculated	Reported
Volume	367.100(4)	367.100(8)
Space group	R 3 c	R 3 c
Hall group	R 3 -2"c	?
Moiety formula	Bi Cr Fe O6 Sr	?
Sum formula	Bi Cr Fe O6 Sr	Bi0.17 Cr0.17 Fe0.17 O Sr0.17
Mr	500.45	83.41
Dx,g cm-3	6.791	0.000
Z	3	18
Mu (mm-1)	120.536	0.000
F000	657.0	0.0
F000'	643.11	
h,k,lmax	6,6,15	5,3,14
Nref	125[64]	128
Tmin,Tmax		1.000,1.000
Tmin'		

Correction method= # Reported T Limits: Tmin=1.000 Tmax=1.000
AbsCorr = ?

Data completeness= 2.00/1.02 Theta(max)= 59.990

R(reflections)= wR2(reflections)=
S = Npar=

The following ALERTS were generated. Each ALERT has the format
test-name_ALERT_alert-type_alert-level.
Click on the hyperlinks for more details of the test.

Alert level A

```
EXPT010_ALERT_1_A  _exptl_crystal_colour (_pd_char_colour for powder) is missing
            Crystal colour.
            The following tests will not be performed.
            CRYSC_01
DIFF003_ALERT_1_A  _diffrn_measurement_device_type is missing
            Diffractometer make and type. Replaces _diffrn_measurement_type.
RADNT01_ALERT_1_A  The radiation type should contain one of the following
            *  'Cu K\a'
            *  'Mo K\a'
            *  'Ag K\a'
            *  'Ga K\a'
            *  'Co K\a'
            *  neutron
            *  synchrotron
SHFSU01_ALERT_2_A  The absolute value of parameter shift to su ratio > 0.20
            Absolute value of the parameter shift to su ratio given   0.350
            Additional refinement cycles may be required.
PLAT044_ALERT_1_A Calculated and Reported Density Dx  Differ by ..     6.7913 Check
PLAT080_ALERT_2_A Maximum Shift/Error ..........................         0.35 Why ?
PLAT197_ALERT_1_A Missing _cell_measurement_temperature Datum ....     Please Add
PLAT198_ALERT_1_A Missing _diffrn_ambient_temperature   Datum ....     Please Add
PLAT702_ALERT_1_A Angle   Calc   173.83(1), Rep      124.07, Dev..    4976.11 Sigma
              O1      -FE1     -SR       104_543  1_555  4_555     #    6 Check
PLAT702_ALERT_1_A Angle   Calc    18.43(1), Rep      126.32, Dev..   10788.97 Sigma
              O1      -FE1     -SR       104_543  1_555104_444     #    7 Check
PLAT702_ALERT_1_A Angle   Calc    10.65(1), Rep       55.01, Dev..    4435.51 Sigma
              O1      -FE1     -SR       104_543  1_555104_544     #    8 Check
PLAT702_ALERT_1_A Angle   Calc    10.56(1), Rep       54.35, Dev..    4379.13 Sigma
              O1      -FE1     -SR       104_543  1_555104_554     #    9 Check
PLAT702_ALERT_1_A Angle   Calc   112.36(1), Rep      125.52, Dev..    1315.60 Sigma
              O1      -FE1     -SR       104_543  1_555201_445     #   10 Check
PLAT702_ALERT_1_A Angle   Calc   112.28(1), Rep      124.86, Dev..    1258.21 Sigma
              O1      -FE1     -SR       104_543  1_555201_455     #   11 Check
PLAT702_ALERT_1_A Angle   Calc   103.17(1), Rep       53.40, Dev..    4976.76 Sigma
              O1      -FE1     -SR       104_543  1_555201_555     #   12 Check
PLAT702_ALERT_1_A Angle   Calc   173.83(1), Rep      124.07, Dev..    4976.11 Sigma
              O1      -FE1     -SR       105_553  1_555  4_555     #   17 Check
PLAT702_ALERT_1_A Angle   Calc    10.56(1), Rep       54.35, Dev..    4379.13 Sigma
              O1      -FE1     -SR       105_553  1_555104_444     #   18 Check
PLAT702_ALERT_1_A Angle   Calc    18.43(1), Rep      126.32, Dev..   10788.97 Sigma
              O1      -FE1     -SR       105_553  1_555104_544     #   19 Check
PLAT702_ALERT_1_A Angle   Calc    10.65(1), Rep       55.01, Dev..    4435.51 Sigma
              O1      -FE1     -SR       105_553  1_555104_554     #   20 Check
PLAT702_ALERT_1_A Angle   Calc   112.28(1), Rep      124.86, Dev..    1258.21 Sigma
              O1      -FE1     -SR       105_553  1_555201_445     #   21 Check
PLAT702_ALERT_1_A Angle   Calc   103.17(1), Rep       53.40, Dev..    4976.76 Sigma
              O1      -FE1     -SR       105_553  1_555201_455     #   22 Check
PLAT702_ALERT_1_A Angle   Calc   112.36(1), Rep      125.52, Dev..    1315.60 Sigma
              O1      -FE1     -SR       105_553  1_555201_555     #   23 Check
PLAT702_ALERT_1_A Angle   Calc   173.83(1), Rep      124.07, Dev..    4976.11 Sigma
```

```
              O1      -FE1     -SR       106_443  1_555  4_555     #   27 Check
PLAT702_ALERT_1_A Angle   Calc    10.65(1), Rep       55.01, Dev..    4435.51 Sigma
              O1      -FE1     -SR       106_443  1_555104_444     #   28 Check
PLAT702_ALERT_1_A Angle   Calc    10.56(1), Rep       54.35, Dev..    4379.13 Sigma
              O1      -FE1     -SR       106_443  1_555104_544     #   29 Check
PLAT702_ALERT_1_A Angle   Calc    18.43(1), Rep      126.32, Dev..   10788.97 Sigma
              O1      -FE1     -SR       106_443  1_555104_554     #   30 Check
PLAT702_ALERT_1_A Angle   Calc   103.17(1), Rep       53.40, Dev..    4976.76 Sigma
              O1      -FE1     -SR       106_443  1_555201_445     #   31 Check
PLAT702_ALERT_1_A Angle   Calc   112.36(1), Rep      125.52, Dev..    1315.60 Sigma
              O1      -FE1     -SR       106_443  1_555201_455     #   32 Check
PLAT702_ALERT_1_A Angle   Calc   112.28(1), Rep      124.86, Dev..    1258.21 Sigma
              O1      -FE1     -SR       106_443  1_555201_555     #   33 Check
PLAT702_ALERT_1_A Angle   Calc   131.79(1), Rep      125.64, Dev..     615.07 Sigma
              O1      -FE1     -SR       201_444  1_555104_444     #   37 Check
PLAT702_ALERT_1_A Angle   Calc   114.13(1), Rep       56.01, Dev..    5811.97 Sigma
              O1      -FE1     -SR       201_444  1_555104_544     #   38 Check
PLAT702_ALERT_1_A Angle   Calc   131.63(1), Rep      125.02, Dev..     661.20 Sigma
              O1      -FE1     -SR       201_444  1_555104_554     #   39 Check
PLAT702_ALERT_1_A Angle   Calc   131.63(1), Rep      125.02, Dev..     661.20 Sigma
              O1      -FE1     -SR       202_554  1_555104_444     #   45 Check
PLAT702_ALERT_1_A Angle   Calc   131.79(1), Rep      125.64, Dev..     615.07 Sigma
              O1      -FE1     -SR       202_554  1_555104_544     #   46 Check
PLAT702_ALERT_1_A Angle   Calc   114.13(1), Rep       56.01, Dev..    5811.97 Sigma
              O1      -FE1     -SR       202_554  1_555104_554     #   47 Check
PLAT702_ALERT_1_A Angle   Calc   114.13(1), Rep       56.01, Dev..    5811.97 Sigma
              O1      -FE1     -SR       203_454  1_555104_444     #   52 Check
PLAT702_ALERT_1_A Angle   Calc   131.63(1), Rep      125.02, Dev..     661.20 Sigma
              O1      -FE1     -SR       203_454  1_555104_544     #   53 Check
PLAT702_ALERT_1_A Angle   Calc   131.79(1), Rep      125.64, Dev..     615.07 Sigma
              O1      -FE1     -SR       203_454  1_555104_554     #   54 Check
PLAT702_ALERT_1_A Angle   Calc   167.73(1), Rep      109.61, Dev..    5812.00 Sigma
              SR      -FE1     -SR         4_555  1_555104_444     #   58 Check
PLAT702_ALERT_1_A Angle   Calc   167.73(1), Rep      109.61, Dev..    5812.00 Sigma
              SR      -FE1     -SR         4_555  1_555104_544     #   59 Check
PLAT702_ALERT_1_A Angle   Calc   167.73(1), Rep      109.61, Dev..    5812.00 Sigma
              SR      -FE1     -SR         4_555  1_555104_554     #   60 Check
PLAT702_ALERT_1_A Angle   Calc    21.21(1), Rep      109.33, Dev..    8812.21 Sigma
              SR      -FE1     -SR       104_444  1_555104_544     #   64 Check
PLAT702_ALERT_1_A Angle   Calc    21.21(1), Rep      109.33, Dev..    8812.21 Sigma
              SR      -FE1     -SR       104_444  1_555104_554     #   65 Check
PLAT702_ALERT_1_A Angle   Calc   102.90(1), Rep       70.53, Dev..    3237.15 Sigma
              SR      -FE1     -SR       104_444  1_555201_445     #   66 Check
PLAT702_ALERT_1_A Angle   Calc   102.90(1), Rep       70.53, Dev..    3237.15 Sigma
              SR      -FE1     -SR       104_444  1_555201_455     #   67 Check
PLAT702_ALERT_1_A Angle   Calc    21.21(1), Rep      109.33, Dev..    8812.21 Sigma
              SR      -FE1     -SR       104_544  1_555104_554     #   69 Check
PLAT702_ALERT_1_A Angle   Calc   102.90(1), Rep       70.53, Dev..    3237.15 Sigma
              SR      -FE1     -SR       104_544  1_555201_445     #   70 Check
PLAT702_ALERT_1_A Angle   Calc   102.90(1), Rep       70.53, Dev..    3237.15 Sigma
              SR      -FE1     -SR       104_544  1_555201_555     #   72 Check
PLAT702_ALERT_1_A Angle   Calc   102.90(1), Rep       70.53, Dev..    3237.15 Sigma
              SR      -FE1     -SR       104_554  1_555201_455     #   74 Check
PLAT702_ALERT_1_A Angle   Calc   102.90(1), Rep       70.53, Dev..    3237.15 Sigma
              SR      -FE1     -SR       104_554  1_555201_555     #   75 Check
PLAT702_ALERT_1_A Angle   Calc    45.51(1), Rep       89.98, Dev..    4446.74 Sigma
              FE1     -O1      -SR       101_555  1_555104_555     #   82 Check
```

```
PLAT702_ALERT_1_A Angle   Calc   132.16(1), Rep       92.35, Dev..    3980.99 Sigma
              FE1     -O1      -SR       204_555  1_555104_555     #   88 Check
PLAT702_ALERT_1_A Angle   Calc    45.51(1), Rep       89.98, Dev..    4446.74 Sigma
              SR      -O1      -CR       104_555  1_555101_555     #  102 Check
PLAT702_ALERT_1_A Angle   Calc   132.16(1), Rep       92.35, Dev..    3980.99 Sigma
              SR      -O1      -CR       104_555  1_555204_555     #  103 Check
PLAT702_ALERT_1_A Angle   Calc    15.79(1), Rep      125.26, Dev..   10947.44 Sigma
              FE1     -SR      -SR         4_554  1_555104_444     #  125 Check
PLAT702_ALERT_1_A Angle   Calc    15.79(1), Rep      125.26, Dev..   10947.44 Sigma
              FE1     -SR      -SR         4_554  1_555104_544     #  126 Check
PLAT702_ALERT_1_A Angle   Calc    15.79(1), Rep      125.26, Dev..   10947.44 Sigma
              FE1     -SR      -SR         4_554  1_555104_554     #  127 Check
PLAT702_ALERT_1_A Angle   Calc    54.88(1), Rep       54.60, Dev..      28.35 Sigma
              FE1     -SR      -SR       101_444  1_555104_444     #  148 Check
PLAT702_ALERT_1_A Angle   Calc    79.04(1), Rep      125.19, Dev..    4615.47 Sigma
              FE1     -SR      -SR       101_444  1_555104_544     #  149 Check
PLAT702_ALERT_1_A Angle   Calc    79.04(1), Rep      125.19, Dev..    4615.47 Sigma
              FE1     -SR      -SR       101_444  1_555104_554     #  150 Check
PLAT702_ALERT_1_A Angle   Calc    79.04(1), Rep      125.19, Dev..    4615.48 Sigma
              FE1     -SR      -SR       101_544  1_555104_444     #  170 Check
PLAT702_ALERT_1_A Angle   Calc    54.88(1), Rep       54.60, Dev..      28.35 Sigma
              FE1     -SR      -SR       101_544  1_555104_544     #  171 Check
PLAT702_ALERT_1_A Angle   Calc    79.04(1), Rep      125.19, Dev..    4615.48 Sigma
              FE1     -SR      -SR       101_544  1_555104_554     #  172 Check
PLAT702_ALERT_1_A Angle   Calc    79.04(1), Rep      125.19, Dev..    4615.47 Sigma
              FE1     -SR      -SR       101_554  1_555104_444     #  191 Check
PLAT702_ALERT_1_A Angle   Calc    79.04(1), Rep      125.19, Dev..    4615.47 Sigma
              FE1     -SR      -SR       101_554  1_555104_544     #  192 Check
PLAT702_ALERT_1_A Angle   Calc    54.88(1), Rep       54.60, Dev..      28.35 Sigma
              FE1     -SR      -SR       101_554  1_555104_554     #  193 Check
PLAT702_ALERT_1_A Angle   Calc   101.23(1), Rep       54.67, Dev..    4656.37 Sigma
              FE1     -SR      -SR       204_444  1_555104_444     #  211 Check
PLAT702_ALERT_1_A Angle   Calc   101.23(1), Rep       54.67, Dev..    4656.37 Sigma
              FE1     -SR      -SR       204_444  1_555104_544     #  212 Check
PLAT702_ALERT_1_A Angle   Calc   125.41(1), Rep      125.13, Dev..      28.44 Sigma
              FE1     -SR      -SR       204_444  1_555104_554     #  213 Check
PLAT702_ALERT_1_A Angle   Calc   101.23(1), Rep       54.67, Dev..    4656.37 Sigma
              FE1     -SR      -SR       204_454  1_555104_444     #  230 Check
PLAT702_ALERT_1_A Angle   Calc   125.41(1), Rep      125.13, Dev..      28.44 Sigma
              FE1     -SR      -SR       204_454  1_555104_544     #  231 Check
PLAT702_ALERT_1_A Angle   Calc   101.23(1), Rep       54.67, Dev..    4656.37 Sigma
              FE1     -SR      -SR       204_454  1_555104_554     #  232 Check
PLAT702_ALERT_1_A Angle   Calc   125.41(1), Rep      125.13, Dev..      28.44 Sigma
              FE1     -SR      -SR       204_554  1_555104_444     #  248 Check
PLAT702_ALERT_1_A Angle   Calc   101.23(1), Rep       54.67, Dev..    4656.37 Sigma
              FE1     -SR      -SR       204_554  1_555104_544     #  249 Check
PLAT702_ALERT_1_A Angle   Calc   101.23(1), Rep       54.67, Dev..    4656.37 Sigma
              FE1     -SR      -SR       204_554  1_555104_554     #  250 Check
PLAT702_ALERT_1_A Angle   Calc    77.86(1), Rep       44.04, Dev..    3382.28 Sigma
              O1      -SR      -SR         1_444  1_555104_444     #  265 Check
PLAT702_ALERT_1_A Angle   Calc   105.13(1), Rep      134.01, Dev..    2888.45 Sigma
              O1      -SR      -SR         1_444  1_555104_554     #  267 Check
PLAT702_ALERT_1_A Angle   Calc   105.12(1), Rep      134.02, Dev..    2890.23 Sigma
              O1      -SR      -SR         1_554  1_555104_444     #  281 Check
PLAT702_ALERT_1_A Angle   Calc    77.85(1), Rep       44.04, Dev..    3380.53 Sigma
              O1      -SR      -SR         1_554  1_555104_554     #  283 Check
PLAT702_ALERT_1_A Angle   Calc    77.85(1), Rep       44.04, Dev..    3380.53 Sigma
```

```
              O1      -SR      -SR         2_554  1_555104_444     #  296 Check
PLAT702_ALERT_1_A Angle   Calc   105.12(1), Rep      134.02, Dev..    2890.23 Sigma
              O1      -SR      -SR         2_554  1_555104_544     #  297 Check
PLAT702_ALERT_1_A Angle   Calc   105.13(1), Rep      134.01, Dev..    2888.45 Sigma
              O1      -SR      -SR         2_654  1_555104_444     #  310 Check
PLAT702_ALERT_1_A Angle   Calc    77.86(1), Rep       44.04, Dev..    3382.28 Sigma
              O1      -SR      -SR         2_654  1_555104_544     #  311 Check
PLAT702_ALERT_1_A Angle   Calc    77.85(1), Rep       44.04, Dev..    3380.53 Sigma
              O1      -SR      -SR         3_554  1_555104_544     #  324 Check
PLAT702_ALERT_1_A Angle   Calc   105.12(1), Rep      134.02, Dev..    2890.24 Sigma
              O1      -SR      -SR         3_554  1_555104_554     #  325 Check
PLAT702_ALERT_1_A Angle   Calc   105.13(1), Rep      134.01, Dev..    2888.45 Sigma
              O1      -SR      -SR         3_564  1_555104_544     #  336 Check
PLAT702_ALERT_1_A Angle   Calc    77.86(1), Rep       44.04, Dev..    3382.28 Sigma
              O1      -SR      -SR         3_564  1_555104_554     #  337 Check
PLAT702_ALERT_1_A Angle   Calc    23.80(1), Rep       88.88, Dev..    6508.19 Sigma
              O1      -SR      -SR       104_543  1_555104_444     #  346 Check
PLAT702_ALERT_1_A Angle   Calc    13.69(1), Rep       45.23, Dev..    3154.24 Sigma
              O1      -SR      -SR       104_543  1_555104_544     #  347 Check
PLAT702_ALERT_1_A Angle   Calc    13.58(1), Rep       44.79, Dev..    3120.94 Sigma
              O1      -SR      -SR       104_543  1_555104_554     #  348 Check
PLAT702_ALERT_1_A Angle   Calc    59.06(1), Rep      135.21, Dev..    7615.06 Sigma
              O1      -SR      -SR       104_543  1_555204_444     #  349 Check
PLAT702_ALERT_1_A Angle   Calc    58.97(1), Rep      134.77, Dev..    7579.76 Sigma
              O1      -SR      -SR       104_543  1_555204_454     #  350 Check
PLAT702_ALERT_1_A Angle   Calc    46.73(1), Rep       91.12, Dev..    4438.81 Sigma
              O1      -SR      -SR       104_543  1_555204_554     #  351 Check
PLAT702_ALERT_1_A Angle   Calc    13.58(1), Rep       44.79, Dev..    3120.94 Sigma
              O1      -SR      -SR       105_553  1_555104_444     #  356 Check
PLAT702_ALERT_1_A Angle   Calc    23.80(1), Rep       88.88, Dev..    6508.19 Sigma
              O1      -SR      -SR       105_553  1_555104_544     #  357 Check
PLAT702_ALERT_1_A Angle   Calc    13.69(1), Rep       45.23, Dev..    3154.24 Sigma
              O1      -SR      -SR       105_553  1_555104_554     #  358 Check
PLAT702_ALERT_1_A Angle   Calc    58.97(1), Rep      134.77, Dev..    7579.76 Sigma
              O1      -SR      -SR       105_553  1_555204_444     #  359 Check
PLAT702_ALERT_1_A Angle   Calc    46.73(1), Rep       91.12, Dev..    4438.81 Sigma
              O1      -SR      -SR       105_553  1_555204_454     #  360 Check
PLAT702_ALERT_1_A Angle   Calc    59.06(1), Rep      135.21, Dev..    7615.06 Sigma
              O1      -SR      -SR       105_553  1_555204_554     #  361 Check
PLAT702_ALERT_1_A Angle   Calc    13.69(1), Rep       45.23, Dev..    3154.24 Sigma
              O1      -SR      -SR       106_443  1_555104_444     #  365 Check
PLAT702_ALERT_1_A Angle   Calc    13.58(1), Rep       44.79, Dev..    3120.94 Sigma
              O1      -SR      -SR       106_443  1_555104_544     #  366 Check
PLAT702_ALERT_1_A Angle   Calc    23.80(1), Rep       88.88, Dev..    6508.19 Sigma
              O1      -SR      -SR       106_443  1_555104_554     #  367 Check
PLAT702_ALERT_1_A Angle   Calc    46.73(1), Rep       91.12, Dev..    4438.81 Sigma
              O1      -SR      -SR       106_443  1_555204_444     #  368 Check
PLAT702_ALERT_1_A Angle   Calc    59.06(1), Rep      135.21, Dev..    7615.06 Sigma
              O1      -SR      -SR       106_443  1_555204_454     #  369 Check
PLAT702_ALERT_1_A Angle   Calc    58.97(1), Rep      134.77, Dev..    7579.76 Sigma
              O1      -SR      -SR       106_443  1_555204_554     #  370 Check
PLAT702_ALERT_1_A Angle   Calc    20.63(1), Rep       88.84, Dev..    6821.33 Sigma
              O1      -SR      -SR       204_443  1_555104_444     #  373 Check
PLAT702_ALERT_1_A Angle   Calc    46.17(1), Rep      135.22, Dev..    8904.74 Sigma
              O1      -SR      -SR       204_443  1_555104_544     #  374 Check
PLAT702_ALERT_1_A Angle   Calc    46.02(1), Rep      134.76, Dev..    8873.92 Sigma
              O1      -SR      -SR       204_443  1_555104_554     #  375 Check
```

```
PLAT702_ALERT_1_A Angle   Calc    46.02(1), Rep      134.76, Dev..    8873.92 Sigma
              O1      -SR      -SR       205_553  1_555104_444     #  380 Check
PLAT702_ALERT_1_A Angle   Calc    20.63(1), Rep       88.84, Dev..    6821.33 Sigma
              O1      -SR      -SR       205_553  1_555104_544     #  381 Check
PLAT702_ALERT_1_A Angle   Calc    46.17(1), Rep      135.22, Dev..    8904.74 Sigma
              O1      -SR      -SR       205_553  1_555104_554     #  382 Check
PLAT702_ALERT_1_A Angle   Calc    46.17(1), Rep      135.22, Dev..    8904.74 Sigma
              O1      -SR      -SR       206_453  1_555104_444     #  386 Check
PLAT702_ALERT_1_A Angle   Calc    46.02(1), Rep      134.76, Dev..    8873.92 Sigma
              O1      -SR      -SR       206_453  1_555104_544     #  387 Check
PLAT702_ALERT_1_A Angle   Calc    20.63(1), Rep       88.84, Dev..    6821.33 Sigma
              O1      -SR      -SR       206_453  1_555104_554     #  388 Check
PLAT702_ALERT_1_A Angle   Calc    27.27(1), Rep       90.00, Dev..    6273.00 Sigma
              SR      -SR      -SR       104_444  1_555104_544     #  392 Check
PLAT702_ALERT_1_A Angle   Calc    27.27(1), Rep       90.00, Dev..    6273.00 Sigma
              SR      -SR      -SR       104_444  1_555104_554     #  393 Check
PLAT702_ALERT_1_A Angle   Calc    48.19(1), Rep       90.00, Dev..    4181.00 Sigma
              SR      -SR      -SR       104_444  1_555204_444     #  394 Check
PLAT702_ALERT_1_A Angle   Calc    48.19(1), Rep       90.00, Dev..    4181.00 Sigma
              SR      -SR      -SR       104_444  1_555204_454     #  395 Check
PLAT702_ALERT_1_A Angle   Calc    27.27(1), Rep       90.00, Dev..    6273.00 Sigma
              SR      -SR      -SR       104_544  1_555104_554     #  397 Check
PLAT702_ALERT_1_A Angle   Calc    48.19(1), Rep       90.00, Dev..    4181.00 Sigma
              SR      -SR      -SR       104_544  1_555204_444     #  398 Check
PLAT702_ALERT_1_A Angle   Calc    48.19(1), Rep       90.00, Dev..    4181.00 Sigma
              SR      -SR      -SR       104_544  1_555204_554     #  400 Check
PLAT702_ALERT_1_A Angle   Calc    48.19(1), Rep       90.00, Dev..    4181.00 Sigma
              SR      -SR      -SR       104_554  1_555204_454     #  402 Check
PLAT702_ALERT_1_A Angle   Calc    48.19(1), Rep       90.00, Dev..    4181.00 Sigma
              SR      -SR      -SR       104_554  1_555204_554     #  403 Check
PLAT702_ALERT_1_A Angle   Calc     6.17(1), Rep       55.93, Dev..    4976.10 Sigma
              O1      -CR      -SR       104_543  1_555  1_555     #  412 Check
PLAT702_ALERT_1_A Angle   Calc   173.83(1), Rep      124.07, Dev..    4976.11 Sigma
              O1      -CR      -SR       104_543  1_555  4_555     #  413 Check
PLAT702_ALERT_1_A Angle   Calc    18.43(1), Rep      126.32, Dev..   10788.97 Sigma
              O1      -CR      -SR       104_543  1_555104_444     #  414 Check
PLAT702_ALERT_1_A Angle   Calc    10.65(1), Rep       55.01, Dev..    4435.51 Sigma
              O1      -CR      -SR       104_543  1_555104_544     #  415 Check
PLAT702_ALERT_1_A Angle   Calc    10.56(1), Rep       54.35, Dev..    4379.13 Sigma
              O1      -CR      -SR       104_543  1_555104_554     #  416 Check
PLAT702_ALERT_1_A Angle   Calc   112.36(1), Rep      125.52, Dev..    1315.60 Sigma
              O1      -CR      -SR       104_543  1_555201_445     #  417 Check
PLAT702_ALERT_1_A Angle   Calc   112.28(1), Rep      124.86, Dev..    1258.21 Sigma
              O1      -CR      -SR       104_543  1_555201_455     #  418 Check
PLAT702_ALERT_1_A Angle   Calc   103.17(1), Rep       53.40, Dev..    4976.76 Sigma
              O1      -CR      -SR       104_543  1_555201_555     #  419 Check
PLAT702_ALERT_1_A Angle   Calc     6.17(1), Rep       55.93, Dev..    4976.10 Sigma
              O1      -CR      -SR       105_553  1_555  1_555     #  424 Check
PLAT702_ALERT_1_A Angle   Calc   173.83(1), Rep      124.07, Dev..    4976.11 Sigma
              O1      -CR      -SR       105_553  1_555  4_555     #  425 Check
PLAT702_ALERT_1_A Angle   Calc    10.56(1), Rep       54.35, Dev..    4379.13 Sigma
              O1      -CR      -SR       105_553  1_555104_444     #  426 Check
PLAT702_ALERT_1_A Angle   Calc    18.43(1), Rep      126.32, Dev..   10788.97 Sigma
              O1      -CR      -SR       105_553  1_555104_544     #  427 Check
PLAT702_ALERT_1_A Angle   Calc    10.65(1), Rep       55.01, Dev..    4435.51 Sigma
              O1      -CR      -SR       105_553  1_555104_554     #  428 Check
PLAT702_ALERT_1_A Angle   Calc   112.28(1), Rep      124.86, Dev..    1258.21 Sigma
```

```
              O1      -CR      -SR       105_553  1_555201_445     #  429 Check
PLAT702_ALERT_1_A Angle   Calc   103.17(1), Rep       53.40, Dev..    4976.76 Sigma
              O1      -CR      -SR       105_553  1_555201_455     #  430 Check
PLAT702_ALERT_1_A Angle   Calc   112.36(1), Rep      125.52, Dev..    1315.60 Sigma
              O1      -CR      -SR       105_553  1_555201_555     #  431 Check
PLAT702_ALERT_1_A Angle   Calc     6.17(1), Rep       55.93, Dev..    4976.10 Sigma
              O1      -CR      -SR       106_443  1_555  1_555     #  435 Check
PLAT702_ALERT_1_A Angle   Calc   173.83(1), Rep      124.07, Dev..    4976.11 Sigma
              O1      -CR      -SR       106_443  1_555  4_555     #  436 Check
PLAT702_ALERT_1_A Angle   Calc    10.65(1), Rep       55.01, Dev..    4435.51 Sigma
              O1      -CR      -SR       106_443  1_555104_444     #  437 Check
PLAT702_ALERT_1_A Angle   Calc    10.56(1), Rep       54.35, Dev..    4379.13 Sigma
              O1      -CR      -SR       106_443  1_555104_544     #  438 Check
PLAT702_ALERT_1_A Angle   Calc    18.43(1), Rep      126.32, Dev..   10788.97 Sigma
              O1      -CR      -SR       106_443  1_555104_554     #  439 Check
PLAT702_ALERT_1_A Angle   Calc   103.17(1), Rep       53.40, Dev..    4976.76 Sigma
              O1      -CR      -SR       106_443  1_555201_445     #  440 Check
PLAT702_ALERT_1_A Angle   Calc   112.36(1), Rep      125.52, Dev..    1315.60 Sigma
              O1      -CR      -SR       106_443  1_555201_455     #  441 Check
PLAT702_ALERT_1_A Angle   Calc   112.28(1), Rep      124.86, Dev..    1258.21 Sigma
              O1      -CR      -SR       106_443  1_555201_555     #  442 Check
PLAT702_ALERT_1_A Angle   Calc   131.79(1), Rep      125.64, Dev..     615.07 Sigma
              O1      -CR      -SR       201_444  1_555104_444     #  447 Check
PLAT702_ALERT_1_A Angle   Calc   114.13(1), Rep       56.01, Dev..    5811.97 Sigma
              O1      -CR      -SR       201_444  1_555104_544     #  448 Check
PLAT702_ALERT_1_A Angle   Calc   131.63(1), Rep      125.02, Dev..     661.20 Sigma
              O1      -CR      -SR       201_444  1_555104_554     #  449 Check
PLAT702_ALERT_1_A Angle   Calc   131.63(1), Rep      125.02, Dev..     661.20 Sigma
              O1      -CR      -SR       202_554  1_555104_444     #  456 Check
PLAT702_ALERT_1_A Angle   Calc   131.79(1), Rep      125.64, Dev..     615.07 Sigma
              O1      -CR      -SR       202_554  1_555104_544     #  457 Check
PLAT702_ALERT_1_A Angle   Calc   114.13(1), Rep       56.01, Dev..    5811.97 Sigma
              O1      -CR      -SR       202_554  1_555104_554     #  458 Check
PLAT702_ALERT_1_A Angle   Calc   114.13(1), Rep       56.01, Dev..    5811.97 Sigma
              O1      -CR      -SR       203_454  1_555104_444     #  464 Check
PLAT702_ALERT_1_A Angle   Calc   131.63(1), Rep      125.02, Dev..     661.20 Sigma
              O1      -CR      -SR       203_454  1_555104_544     #  465 Check
PLAT702_ALERT_1_A Angle   Calc   131.79(1), Rep      125.64, Dev..     615.07 Sigma
              O1      -CR      -SR       203_454  1_555104_554     #  466 Check
PLAT702_ALERT_1_A Angle   Calc    12.27(1), Rep       70.39, Dev..    5812.00 Sigma
              SR      -CR      -SR         1_555  1_555104_444     #  471 Check
PLAT702_ALERT_1_A Angle   Calc    12.27(1), Rep       70.39, Dev..    5812.00 Sigma
              SR      -CR      -SR         1_555  1_555104_544     #  472 Check
PLAT702_ALERT_1_A Angle   Calc    12.27(1), Rep       70.39, Dev..    5812.00 Sigma
              SR      -CR      -SR         1_555  1_555104_554     #  473 Check
PLAT702_ALERT_1_A Angle   Calc   167.73(1), Rep      109.61, Dev..    5812.00 Sigma
              SR      -CR      -SR         4_555  1_555104_444     #  477 Check
PLAT702_ALERT_1_A Angle   Calc   167.73(1), Rep      109.61, Dev..    5812.00 Sigma
              SR      -CR      -SR         4_555  1_555104_544     #  478 Check
PLAT702_ALERT_1_A Angle   Calc   167.73(1), Rep      109.61, Dev..    5812.00 Sigma
              SR      -CR      -SR         4_555  1_555104_554     #  479 Check
PLAT702_ALERT_1_A Angle   Calc    21.21(1), Rep      109.33, Dev..    8812.21 Sigma
              SR      -CR      -SR       104_444  1_555104_544     #  483 Check
PLAT702_ALERT_1_A Angle   Calc    21.21(1), Rep      109.33, Dev..    8812.21 Sigma
              SR      -CR      -SR       104_444  1_555104_554     #  484 Check
PLAT702_ALERT_1_A Angle   Calc   102.90(1), Rep       70.53, Dev..    3237.15 Sigma
              SR      -CR      -SR       104_444  1_555201_445     #  485 Check
```

```
PLAT702_ALERT_1_A Angle   Calc   102.90(1), Rep      70.53, Dev..    3237.15 Sigma
              SR      -CR       -SR       104_444  1_555201_455     #  486 Check
PLAT702_ALERT_1_A Angle   Calc    21.21(1), Rep     109.33, Dev..    8812.21 Sigma
              SR      -CR       -SR       104_544  1_555104_554     #  488 Check
PLAT702_ALERT_1_A Angle   Calc   102.90(1), Rep      70.53, Dev..    3237.15 Sigma
              SR      -CR       -SR       104_544  1_555201_445     #  489 Check
PLAT702_ALERT_1_A Angle   Calc   102.90(1), Rep      70.53, Dev..    3237.15 Sigma
              SR      -CR       -SR       104_544  1_555201_555     #  491 Check
PLAT702_ALERT_1_A Angle   Calc   102.90(1), Rep      70.53, Dev..    3237.15 Sigma
              SR      -CR       -SR       104_554  1_555201_455     #  493 Check
PLAT702_ALERT_1_A Angle   Calc   102.90(1), Rep      70.53, Dev..    3237.15 Sigma
              SR      -CR       -SR       104_554  1_555201_555     #  494 Check
PLAT722_ALERT_1_A Angle   Calc   121.60(1), Rep     179.72 Dev...      58.12 Degree
              SR      -FE1      -SR       104_444  1_555201_555     #   68 Check
PLAT722_ALERT_1_A Angle   Calc   121.60(1), Rep     179.72 Dev...      58.12 Degree
              SR      -FE1      -SR       104_544  1_555201_455     #   71 Check
PLAT722_ALERT_1_A Angle   Calc   121.60(1), Rep     179.72 Dev...      58.12 Degree
              SR      -FE1      -SR       104_554  1_555201_445     #   73 Check
PLAT722_ALERT_1_A Angle   Calc    42.23(1), Rep     177.68 Dev...     135.45 Degree
              SR      -O1       -SR       104_555  1_555204_555     #  101 Check
PLAT722_ALERT_1_A Angle   Calc    70.53(1), Rep     180.00 Dev...     109.47 Degree
              SR      -SR       -SR       104_444  1_555204_554     #  396 Check
PLAT722_ALERT_1_A Angle   Calc    70.53(1), Rep     179.97 Dev...     109.44 Degree
              SR      -SR       -SR       104_544  1_555204_454     #  399 Check
PLAT722_ALERT_1_A Angle   Calc    70.53(1), Rep     180.00 Dev...     109.47 Degree
              SR      -SR       -SR       104_554  1_555204_444     #  401 Check
PLAT722_ALERT_1_A Angle   Calc   121.60(1), Rep     179.72 Dev...      58.12 Degree
              SR      -CR       -SR       104_444  1_555201_555     #  487 Check
PLAT722_ALERT_1_A Angle   Calc   121.60(1), Rep     179.72 Dev...      58.12 Degree
              SR      -CR       -SR       104_544  1_555201_455     #  490 Check
PLAT722_ALERT_1_A Angle   Calc   121.60(1), Rep     179.72 Dev...      58.12 Degree
              SR      -CR       -SR       104_554  1_555201_445     #  492 Check
PLAT902_ALERT_1_A No (Interpretable) Reflections Found in FCF ....     Please Check
```

Alert level B

```
PLAT722_ALERT_1_B Angle   Calc    88.46(1), Rep      90.71 Dev...       2.25 Degree
              SR      -O1       -SR         1_556  1_555104_555     #   93 Check
PLAT722_ALERT_1_B Angle   Calc    88.56(1), Rep      91.18 Dev...       2.62 Degree
              SR      -O1       -SR         1_666  1_555104_555     #   97 Check
PLAT722_ALERT_1_B Angle   Calc    91.30(1), Rep      88.86 Dev...       2.44 Degree
              O1      -SR       -SR         1_444  1_555104_544     #  266 Check
PLAT722_ALERT_1_B Angle   Calc    91.29(1), Rep      88.87 Dev...       2.42 Degree
              O1      -SR       -SR         1_554  1_555104_544     #  282 Check
PLAT722_ALERT_1_B Angle   Calc    91.29(1), Rep      88.87 Dev...       2.42 Degree
              O1      -SR       -SR         2_554  1_555104_554     #  298 Check
PLAT722_ALERT_1_B Angle   Calc    91.30(1), Rep      88.86 Dev...       2.44 Degree
              O1      -SR       -SR         2_654  1_555104_554     #  312 Check
PLAT722_ALERT_1_B Angle   Calc    91.29(1), Rep      88.87 Dev...       2.42 Degree
              O1      -SR       -SR         3_554  1_555104_444     #  323 Check
PLAT722_ALERT_1_B Angle   Calc    91.30(1), Rep      88.86 Dev...       2.44 Degree
              O1      -SR       -SR         3_564  1_555104_444     #  335 Check
```

Alert level C

```
CHEMW01_ALERT_1_C  The ratio of given/expected molecular weight as calculated
            from the _chemical_formula_sum lies outside
```

```
            the range 0.99 <> 1.01
            Calculated formula weight =    84.7548
            Formula weight given      =    83.4100
CRYSC01_ALERT_1_C No recognised colour has been given for crystal colour.
PLAT125_ALERT_4_C No '_symmetry_space_group_name_Hall' Given .....     Please Do !
PLAT701_ALERT_1_C Bond    Calc 2.83420(10), Rep  2.83408(2), Dev..       1.20 Sigma
              BI1      -O1              1_555 104_544 ........     #    7 Check
PLAT701_ALERT_1_C Bond    Calc 2.83420(10), Rep  2.83408(2), Dev..       1.20 Sigma
              BI1      -O1              1_555 105_554 ........     #    8 Check
PLAT701_ALERT_1_C Bond    Calc 2.83420(10), Rep  2.83408(2), Dev..       1.20 Sigma
              BI1      -O1              1_555 106_444 ........     #    9 Check
PLAT701_ALERT_1_C Bond    Calc 3.42140(10), Rep  3.42129(3), Dev..       1.10 Sigma
              BI1      -CR              1_555   1_555 ........     #   19 Check
PLAT701_ALERT_1_C Bond    Calc 3.40360(10), Rep  3.40374(3), Dev..       1.40 Sigma
              BI1      -CR              1_555   4_554 ........     #   20 Check
PLAT701_ALERT_1_C Bond    Calc 3.42140(10), Rep  3.42129(3), Dev..       1.10 Sigma
              FE1      -SR              1_555   1_555 ........     #   33 Check
PLAT701_ALERT_1_C Bond    Calc 3.40360(10), Rep  3.40374(3), Dev..       1.40 Sigma
              FE1      -SR              1_555   4_555 ........     #   34 Check
PLAT701_ALERT_1_C Bond    Calc 2.83420(10), Rep  2.83408(2), Dev..       1.20 Sigma
              O1       -BI1             1_555 204_555 ........     #   44 Check
PLAT701_ALERT_1_C Bond    Calc 2.83420(10), Rep  2.83408(2), Dev..       1.20 Sigma
              O1       -SR              1_555 204_555 ........     #   50 Check
PLAT701_ALERT_1_C Bond    Calc 3.42140(10), Rep  3.42129(3), Dev..       1.10 Sigma
              SR       -FE1             1_555   1_555 ........     #   59 Check
PLAT701_ALERT_1_C Bond    Calc 3.40360(10), Rep  3.40374(3), Dev..       1.40 Sigma
              SR       -FE1             1_555   4_554 ........     #   60 Check
PLAT701_ALERT_1_C Bond    Calc 2.83420(10), Rep  2.83408(2), Dev..       1.20 Sigma
              SR       -O1              1_555 104_544 ........     #   73 Check
PLAT701_ALERT_1_C Bond    Calc 2.83420(10), Rep  2.83408(2), Dev..       1.20 Sigma
              SR       -O1              1_555 105_554 ........     #   74 Check
PLAT701_ALERT_1_C Bond    Calc 2.83420(10), Rep  2.83408(2), Dev..       1.20 Sigma
              SR       -O1              1_555 106_444 ........     #   75 Check
PLAT701_ALERT_1_C Bond    Calc 3.42140(10), Rep  3.42129(3), Dev..       1.10 Sigma
              SR       -CR              1_555   1_555 ........     #   85 Check
PLAT701_ALERT_1_C Bond    Calc 3.40360(10), Rep  3.40374(3), Dev..       1.40 Sigma
              SR       -CR              1_555   4_554 ........     #   86 Check
PLAT701_ALERT_1_C Bond    Calc 3.42140(10), Rep  3.42129(3), Dev..       1.10 Sigma
              CR       -BI1             1_555   1_555 ........     #   93 Check
PLAT701_ALERT_1_C Bond    Calc 3.40360(10), Rep  3.40374(3), Dev..       1.40 Sigma
              CR       -BI1             1_555   4_555 ........     #   94 Check
PLAT701_ALERT_1_C Bond    Calc 3.42140(10), Rep  3.42129(3), Dev..       1.10 Sigma
              CR       -SR              1_555   1_555 ........     #  107 Check
PLAT701_ALERT_1_C Bond    Calc 3.40360(10), Rep  3.40374(3), Dev..       1.40 Sigma
              CR       -SR              1_555   4_555 ........     #  108 Check
```

Alert level G

```
FORMU01_ALERT_1_G  There is a discrepancy between the atom counts in the
            _chemical_formula_sum and the formula from the _atom_type* data.
            Atom count from _chemical_formula_sum:Bi0.17 Cr0.17 Fe0.17 O1 Sr0.17
            Atom count from the _atom_type data:  Bi0.166666 Cr0.166666 Fe0.166666
FORMU01_ALERT_2_G  There is a discrepancy between the atom counts in the
            _chemical_formula_sum and the formula from the _atom_site* data.
            Atom count from _chemical_formula_sum:Bi0.17 Cr0.17 Fe0.17 O1 Sr0.17
            Atom count from the _atom_site data:  Bi0.166666 Cr0.166666 Fe0.166666
CELLZ01_ALERT_1_G Difference between formula and atom_site contents detected.
```

```
CELLZ01_ALERT_1_G ALERT: check formula stoichiometry or atom site occupancies.
           From the CIF: _cell_formula_units_Z   18
           From the CIF: _chemical_formula_sum  Bi0.17 Cr0.17 Fe0.17 O Sr0.17
           TEST: Compare cell contents of formula and atom_site data
           WARNING: Unexpected atom type is in site list: Bi
           WARNING: Unexpected atom type is in site list: Fe
           WARNING: Unexpected atom type is in site list: O-
           WARNING: Formula and atom_type_symbol element names mismatch.

           atom    Z*formula  cif sites diff
           Sr         3.06      3.00    0.06
           Cr         3.06      3.00    0.06
           Bi         3.06      3.00    0.06
           Fe         3.06      3.00    0.06
            WARNING: Site labels do not match formula elements
PLAT004_ALERT_5_G Polymeric Structure Found with Maximum Dimension          3 Info
PLAT017_ALERT_1_G Check Scattering Type Consistency of O1       as         O-
PLAT045_ALERT_1_G Calculated and Reported Z Differ by a Factor ...     0.1667 Check
PLAT092_ALERT_4_G Check: Wavelength Given is not Cu,Ga,Mo,Ag,In Ka    1.54430 Ang.
PLAT143_ALERT_4_G s.u. on c - Axis Small or Missing .............    0.00010 Ang.
PLAT152_ALERT_1_G The Supplied and Calc. Volume s.u. Differ by ...         -4 Units
PLAT300_ALERT_4_G Atom Site Occupancy of Bi1       Constrained at        0.5 Check
PLAT300_ALERT_4_G Atom Site Occupancy of Sr        Constrained at        0.5 Check
PLAT300_ALERT_4_G Atom Site Occupancy of Fe1       Constrained at        0.5 Check
PLAT300_ALERT_4_G Atom Site Occupancy of Cr        Constrained at        0.5 Check
PLAT301_ALERT_3_G Main Residue  Disorder .............(Resd  1  )        67% Note
PLAT720_ALERT_4_G Number of Unusual/Non-Standard Labels ..........          1 Note
PLAT769_ALERT_4_G CIF Embedded explicitly supplied scattering data     Please Note
PLAT811_ALERT_5_G No ADDSYM Analysis: Too Many Excluded Atoms ....          ! Info
PLAT981_ALERT_1_G No non-zero f" Anomalous Scattering Values Found     Please Check
PLAT986_ALERT_1_G No non-zero f' Anomalous Scattering Values Found     Please Check
```

---

182 **ALERT level A** = Most likely a serious problem - resolve or explain
8 **ALERT level B** = A potentially serious problem, consider carefully
23 **ALERT level C** = Check. Ensure it is not caused by an omission or oversight
20 **ALERT level G** = General information/check it is not something unexpected

218 ALERT type 1 CIF construction/syntax error, inconsistent or missing data
3 ALERT type 2 Indicator that the structure model may be wrong or deficient
1 ALERT type 3 Indicator that the structure quality may be low
9 ALERT type 4 Improvement, methodology, query or suggestion
2 ALERT type 5 Informative message, check

---

checkCIF publication errors

```
Alert level A
PUBL003_ALERT_1_A  The contact author's name is missing,
            _publ_contact_author_name.
PUBL005_ALERT_1_A  _publ_contact_author_email, _publ_contact_author_fax and
            _publ_contact_author_phone are all missing.
            At least one of these should be present.
PUBL006_ALERT_1_A  _publ_requested_journal is missing
            e.g. 'Acta Crystallographica Section C'
PUBL009_ALERT_1_A  _publ_author_name is missing. List of author(s) name(s).
```

```
   4 ALERT level A = Data missing that is essential or data in wrong format
   0 ALERT level G = General alerts. Data that may be required is missing
```

Publication of your CIF

You should attempt to resolve as many as possible of the alerts in all categories. Often the minor alerts point to easily fixed oversights, errors and omissions in your CIF or refinement strategy, so attention to these fine details can be worthwhile. In order to resolve some of the more serious problems it may be necessary to carry out additional measurements or structure refinements. However, the nature of your study may justify the reported deviations from journal submission requirements and the more serious of these should be commented upon in the discussion or experimental section of a paper or in the "special_details" fields of the CIF. *checkCIF* was carefully designed to identify outliers and unusual parameters, but every test has its limitations and alerts that are not important in a particular case may appear. Conversely, the absence of alerts does not guarantee there are no aspects of the results needing attention. It is up to the individual to critically assess their own results and, if necessary, seek expert advice.

If level A alerts remain, which you believe to be justified deviations, and you intend to submit this CIF for publication in a journal, you should additionally insert an explanation in your CIF using the Validation Reply Form (VRF) below. This will allow your explanation to be considered as part of the review process.

Validation response form

Please find below a validation response form (VRF) that can be filled in and pasted into your CIF.

```
# start Validation Reply Form
_vrf_PUBL003_GLOBAL
;
PROBLEM: The contact author's name is missing,
RESPONSE: ...
;
_vrf_PUBL005_GLOBAL
;
PROBLEM: _publ_contact_author_email, _publ_contact_author_fax and
RESPONSE: ...
;
_vrf_PUBL006_GLOBAL
```

```
;
PROBLEM: _publ_requested_journal is missing
RESPONSE: ...
;
_vrf_PUBL009_GLOBAL
;
PROBLEM: _publ_author_name is missing. List of author(s) name(s).
RESPONSE: ...
;
_vrf_EXPT010_BFSC_publ
;
PROBLEM: _exptl_crystal_colour (_pd_char_colour for powder) is missing
RESPONSE: ...
;
_vrf_DIFF003_BFSC_publ
;
PROBLEM: _diffrn_measurement_device_type is missing
RESPONSE: ...
;
_vrf_RADNT01_BFSC_publ
;
PROBLEM: The radiation type should contain one of the following
RESPONSE: ...
;
_vrf_SHFSU01_BFSC_publ
;
PROBLEM: The absolute value of parameter shift to su ratio > 0.20
RESPONSE: ...
;
_vrf_CHEMW01_BFSC_publ
;
PROBLEM: The ratio of given/expected molecular weight as calculated
RESPONSE: ...
;
_vrf_CRYSC01_BFSC_publ
;
PROBLEM: No recognised colour has been given for crystal colour.
RESPONSE: ...
;
_vrf_PLAT044_BFSC_publ
;
PROBLEM: Calculated and Reported Density Dx  Differ by ..     6.7913 Check
RESPONSE: ...
;
_vrf_PLAT080_BFSC_publ
;
PROBLEM: Maximum Shift/Error ...........................       0.35 Why ?
RESPONSE: ...
;
_vrf_PLAT197_BFSC_publ
;
PROBLEM: Missing _cell_measurement_temperature Datum ....     Please Add
RESPONSE: ...
;
_vrf_PLAT198_BFSC_publ
;
PROBLEM: Missing _diffrn_ambient_temperature   Datum ....     Please Add
```

```
RESPONSE: ...
;
_vrf_PLAT702_BFSC_publ
;
PROBLEM: Angle   Calc   173.83(1), Rep      124.07, Dev..    4976.11 Sigma
RESPONSE: ...
;
_vrf_PLAT722_BFSC_publ
;
PROBLEM: Angle   Calc   121.60(1), Rep      179.72 Dev...      58.12 Degree
RESPONSE: ...
;
_vrf_PLAT902_BFSC_publ
;
PROBLEM: No (Interpretable) Reflections Found in FCF ....     Please Check
RESPONSE: ...
;
_vrf_PLAT125_BFSC_publ
;
PROBLEM: No '_symmetry_space_group_name_Hall' Given .....     Please Do !
RESPONSE: ...
;
_vrf_PLAT701_BFSC_publ
;
PROBLEM: Bond    Calc 2.83420(10), Rep  2.83408(2), Dev..       1.20 Sigma
RESPONSE: ...
;
# end Validation Reply Form
```

If you wish to submit your CIF for publication in Acta Crystallographica Section C or E, you should upload your CIF via the web. If you wish to submit your CIF for publication in IUCrData you should upload your CIF via the web. If your CIF is to form part of a submission to another IUCr journal, you will be asked, either during electronic submission or by the Co-editor handling your paper, to upload your CIF via our web site.

PLATON version of 20/01/2022; check.def file version of 19/01/2022

Datablock BFSC_publ - ellipsoid plot

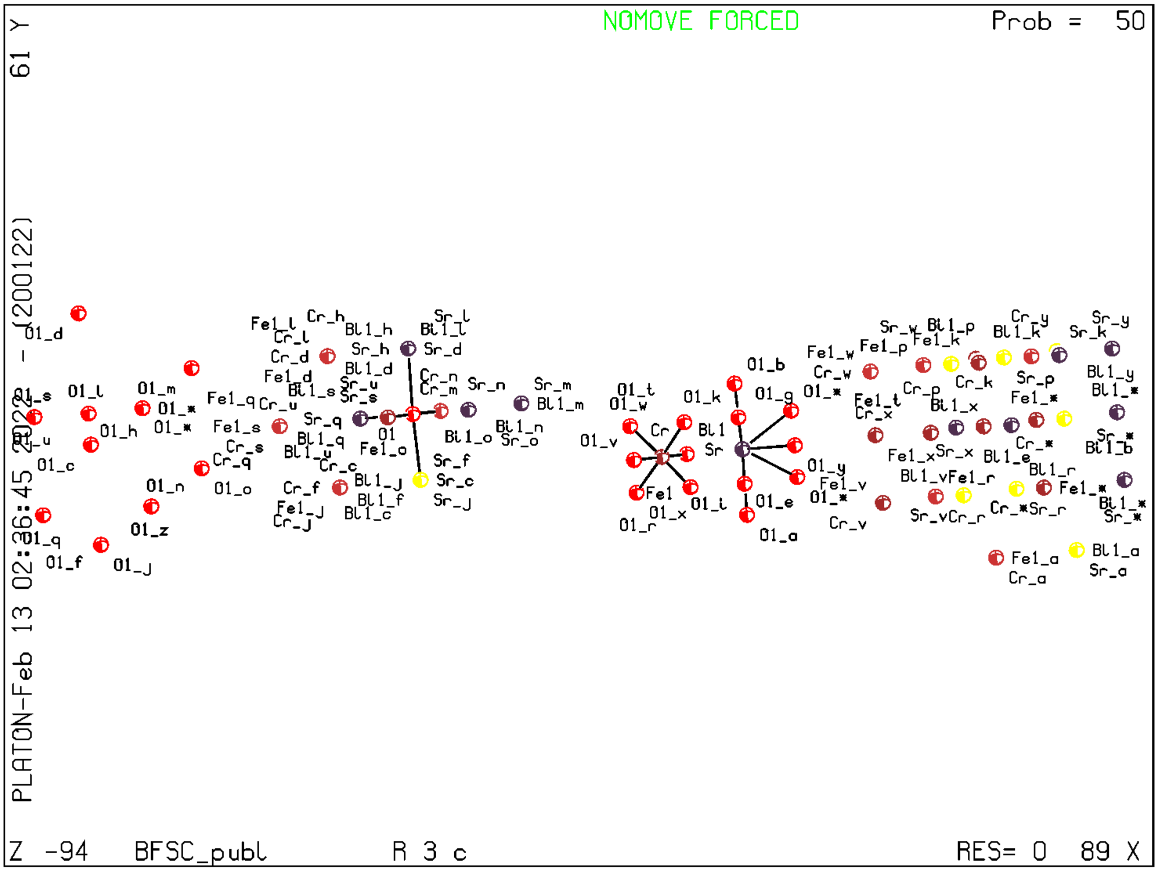

Supplement: Supplementary file 1 — Supplementary Information. [file 41598_2023_32734_MOESM1_ESM.pdf]
